# Supplementary material for: Cooperative interaction between ERα and the EMT-inducer ZEB1 reprograms breast cancer cells for bone metastasis
Source: Nat Commun. 2022 Apr 19;13:2104. doi: 10.1038/s41467-022-29723-5 (PMC9018728; doi:10.1038/s41467-022-29723-5)
Supplement: Supplementary file 1 — Supplementary Information [file 41467_2022_29723_MOESM1_ESM.pdf]

Supplementary Information

Cooperative interaction between ERα and the EMT-inducer ZEB1 reprograms breast cancer cells for bone metastasis

Ghahhari et al.

Supplementary Information includes Supplementary Note 1, Supplementary Methods, eleven Supplementary Figures with legends, four Supplementary Tables, and Supplementary References

Supplementary Note 1

Zinc finger cluster 1 of ZEB1 and F-domain of ERα are required for augmented ERα activity. ZEB1 is composed of two zinc finger clusters (ZF1 and ZF2) with a central homeodomain. ZEB1 uses its ZF1/2 clusters to bind DNA through specific E-boxes[1,2]. To determine which domain(s) of ZEB1 are involved in its interaction with ERα, we generated several truncation mutants (Supplementary Fig. 3a). Full-length ZEB1 induced ERα activity, while the deletion of ZF1 resulted in the loss of ZEB1-induced ERα regulation. In contrast, the truncation of ZF2 did not affect the ZEB1-mediated activation of the ERE-Luc reporter. Double-deletion mutants of the ZF1/2 significantly reduced the impact on ERα activity and the homeodomain of ZEB1 appeared to be dispensable for ERα-augmented activity (Supplementary Fig. 3b). These findings point to the importance of ZF1 of ZEB1 for increasing ERα activity. To further explore the possible involvement of various ZEB1 domains in the physical interaction with ERα, we performed co-IPs and compared the interaction of ZEB1 mutants with full-length ERα. Mirroring our observations with the luciferase assays, ZF1 was required for the ZEB1-ERα interaction. Excision of neither ZF2 nor the homeodomain affected the ERα interaction with ZEB1 (Supplementary Fig. 3c).

Next, we sought to identify the ERα domain(s) that mediate the activation by ZEB1. We used Gal4-ERα fusion proteins, which consist of the Gal4 DNA binding domain fused to the activation function 1 (AF1), the DNA binding domain (DBD), the hormone binding domain (HBD) alone including the AF2, or the HBD with the C-terminal F-domain of ERα (Supplementary Fig. 3d). In response to E2, only the activity of the HBD chimera with the F-domain proved to be stimulated by ZEB1, albeit only modestly, suggesting that the F-domain of ERα plays a key role for the enhancement of ERα activity by ZEB1 (Supplementary Fig. 3e). Indeed, the F-domain modifies the HBD function, and in its absence ERα fails to activate transcription through SP1[3]. We failed to observe a direct interaction of these ZEB1 and ERα domains, suggesting that either a direct interaction requires other domains as well or that the ZEB1-ERα interaction is indirect through another protein(s) in the same complex.

Supplementary Methods

Cell lines. HEK293T and MCF7 cells were purchased from the American Type Culture Collection (ATCC). T-47D cells of the European Collection of Authenticated Cell Cultures (ECACC) were purchased from Sigma Aldrich). MCF7-V cells were a gift from Dr. Wilbert Zwart (Netherlands Cancer Institute, Amsterdam); their highly polymorphic short tandem repeat loci (STRs) were profiled using commercial services (ATCC and Microsynth) and found to be closely (88%) related to wild-type MCF7 cells; specifically, their eight core STR markers were as follows (alleles are indicated in parenthesis): D5S818 (11, 12), D13S317 (11), D7S820 (8, 9), D16S539 (11, 12), vWA (14, 15), TH01 (6), TPOX (9), and CSF1PO (10, 11).

Plasmids. The constructs pTRIPz-ΔZF1-ZEB1, pTRIPz-ΔZF2-ZEB1, pTRIPz-ΔZF1/2-ZEB1, and pTRIPz-ΔHD-ZEB1 for the expression of the corresponding ZEB1 truncation mutants were generated by Gibson assembly (In-Fusion HD Cloning Plus, Takara Bio) with the primers listed in Supplementary Table 4. Briefly, for pTRIPz-ΔZF1-ZEB1, the sequences encoding the zinc finger (ZF) cluster 1 domain were deleted with the assembly of two PCR amplicons corresponding to sequences for aa 1-149 and aa 272-1061 from the full-length ZEB1. HA-ZEB1 fragment was excised by restriction digestion to generate the empty lentiviral vector pTRIPz. To construct pTRIPz-ΔZF2-ZEB1, two PCR fragments corresponding to coding sequences for aa 1-881 and aa 960-1061 were assembled into the empty vector pTRIPz. For plasmid pTRIPz-ΔZF1/2-ZEB1, three fragments were PCR amplified from the full-length ZEB1 (corresponding to sequences for aa 1-149, aa 272-881 and aa 960-1061) and were ligated into the empty vector. Finally, to engineer the construct pTRIPz-ΔHD-ZEB1 for the homeodomain mutant, two PCR amplicons equivalent to the coding sequences for aa 1-560 and aa 619-1061 from full-length ZEB1 were assembled into the vector pTRIPz. The following constructs were used for the Gal4 fusions: plasmid pSCTEV gal93 for the expression of the following fusions with the Gal4 DNA binding domain (DBD, aa 1-93)[4]: Gal93.ER(G) (also referred to as Gal4.ERα) with the ERα HBD (aa 282-595), Gal4.ER(AF1) with AF1 (aa 82-152)[5], Gal4.ER(AF2) with AF2 (aa 282-595), Gal93.ERΔF with the HBD without the F-domain (aa 282-553)[6], and Gal93.ER-DBD with the ERα DBD (aa 180-262)[7]. Luciferase reporter plasmid GK1[8] was used for Gal4 fusion proteins.

Supplementary Figures

Supplementary Fig. 1

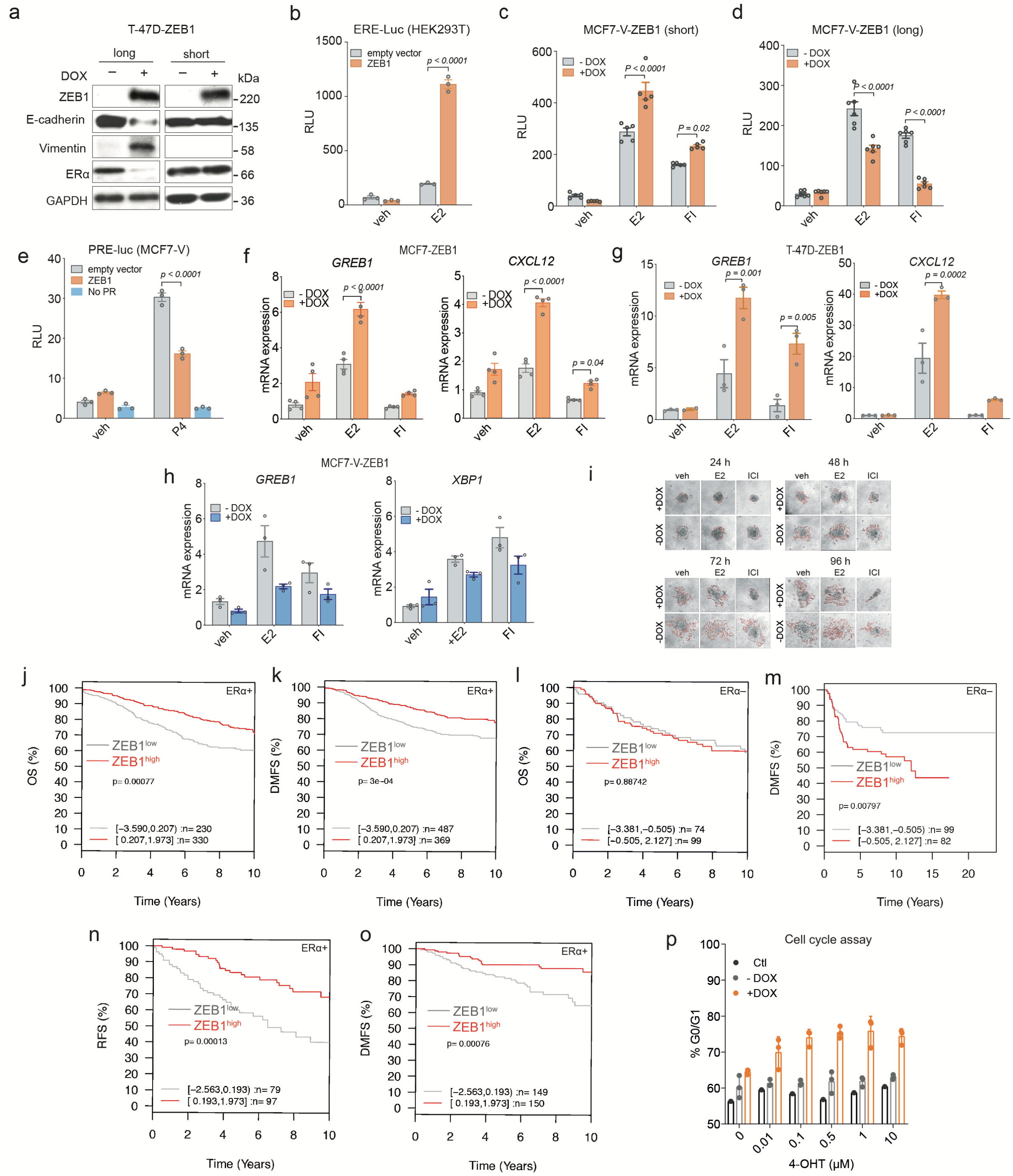

Supplementary Fig. 1 ZEB1 enhances ERα activity and improves breast cancer patient outcome. a T-47D-ZEB1 cells express ZEB1 in the presence of DOX. Immunoblots show E-cadherin and vimentin levels in cells after long-term (8-12 weeks) and short-term (1-2 weeks) expression of ZEB1. **b** Luciferase reporter assays with HEK293T cells transiently transfected with ERE-Luc (n = 3 independent

experiments). **c, d** Luciferase reporter assays with ERE-Luc with MCF7-V-ZEB1 cells showing the quantification of ERα activity with indicated treatments after short– (1 week) and long-term (12-weeks) expression of ZEB1 (n = 5 for panel c and n = 6 for panel d). **e** Luciferase reporter assay with HEK293T cells transiently transfected with the reporter PRE-Luc (n = 3). **f, g** mRNA levels of ERα target genes in MCF7-ZEB1 (**f**) and T-47D-ZEB1 (**g**) cells after induction of ZEB1 expression for one week and following 6 h of treatments as indicated (n = 4 for panel f and n = 3 for panel g). **h** mRNA levels of ERα target genes in MCF7-V-ZEB1 cells upon long-term expression of ZEB1 (+DOX). RT-qPCRs were performed following 6 h of indicated treatments (n = 3). **i** Representative three-dimensional (3D) tumor invasion assays with T-47D-ZEB1 cells treated as indicated (n = 2 biologically independent experiments). **j-m** Kaplan-Meier plots for overall survival (OS) and distant metastasis-free survival (DMFS) of $ER\alpha^{+}$ (**j, k**) and $ER\alpha^{-}$ (**l, m**) breast cancer patients classified as tumors expressing high levels (red line) and low levels (black line) of ZEB1. **n, o** Kaplan-Meier plots for relapse-free survival (RFS) (**n**) and DMFS (**o**) of $ER\alpha^{+}$ breast cancer patients treated with tamoxifen classified as tumors expressing high levels (red line) and low levels (gray line) of ZEB1. **p** Cell cycle assay with MCF7-V-ZEB1 cells after 72 h of treatment with increasing concentration of 4-OHT. veh, E2, FI, and ICI stand for vehicle, 17β-estradiol, forskolin + IBMX, and fulvestrant, respectively (n = 3). All error bars represent standard errors of the means (mean ± SEM). p-values are indicated above the bars; statistical significance was determined with a two-way ANOVA. Source data are provided as a Source Data file.

Supplementary Fig. 2

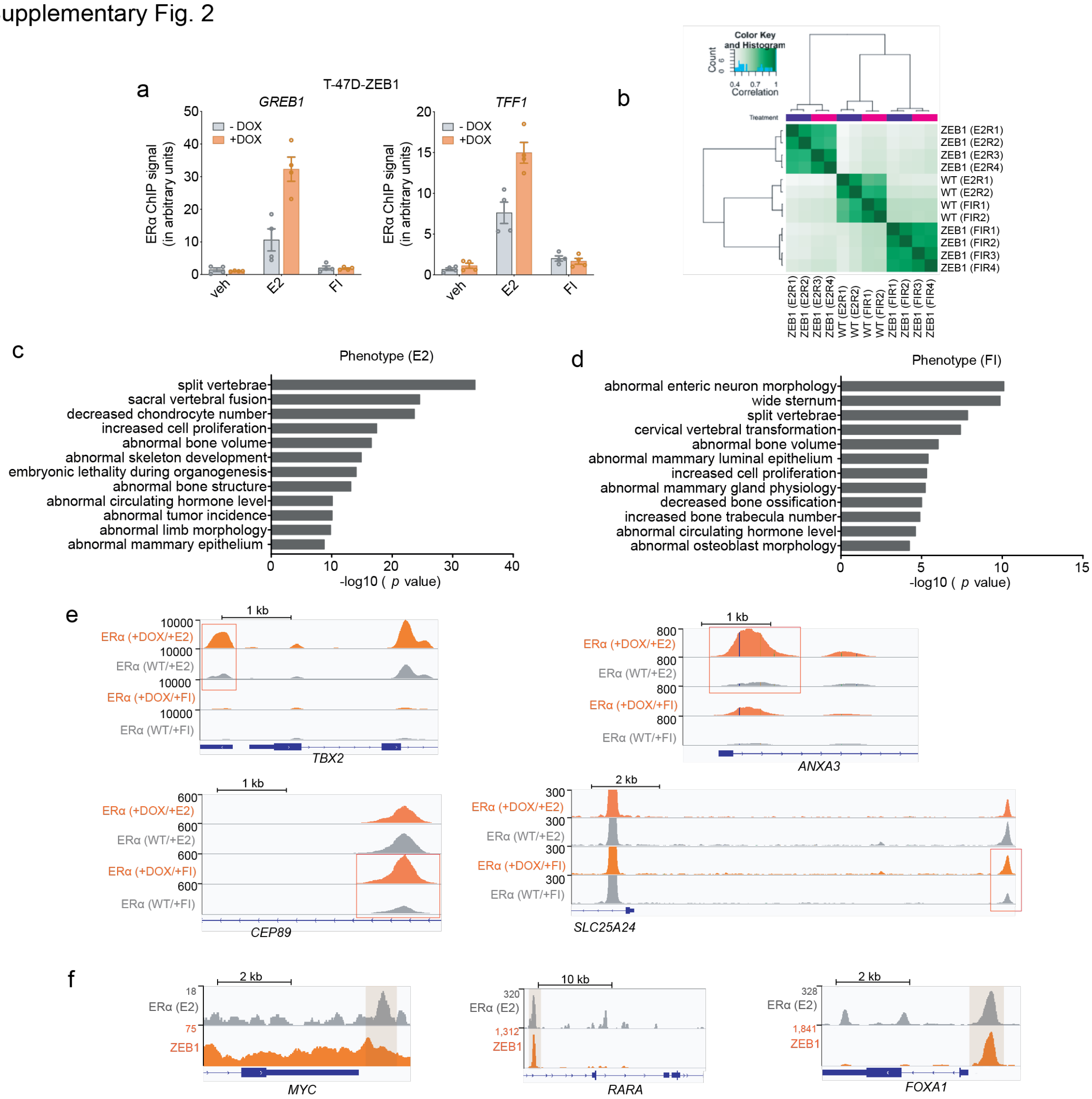

Supplementary Fig. 2 ZEB1 potentiates ERα binding on novel binding sites. a ChIP-qPCR of ERα on the proximal binding sites of the *GREB1* and *TFF1* genes in T-47D-ZEB1 cells. All error bars represent standard errors of the means (mean ± SEM) (n = 4 biologically independent experiments). **b** Correlation heatmap with pairwise correlations and hierarchical clustering of read densities of ERα ChIP-seq replicate samples of WT MCF7-V and MCF7-V-ZEB1 cells. **c, d** Functional annotations for the biological phenotypes of E2– (**c**) or FI-only (**d**) ERα binding sites, using GREAT. **e** Genome bowser views of ERα binding sites adjacent to the *TBX2*, *ANXA3*, *CEP89*, and *SLC25A24* genes. Orange boxes indicate the sites with altered ERα binding in presence of indicated treatments. veh, E2, and FI stand for vehicle, 17β-estradiol, and forskolin + IBMX, respectively. Source data are provided in the Source Data file.

Supplementary Fig. 3

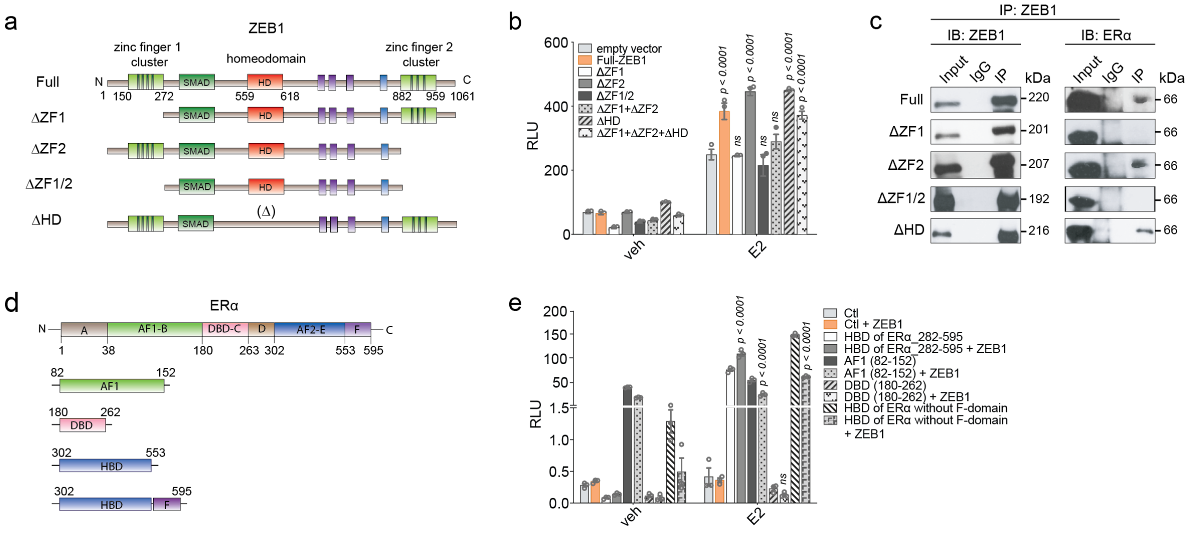

Supplementary Fig. 3 Analyses of ZEB1 and ERα domains in potentiating the ERα activity. **a** Schematic representation of ZEB1 truncation mutants. **b** ERE-luciferase reporter assays were performed upon transient coexpression of ERα with full-length or truncated ZEB1 mutants in HEK293T cells with the indicated treatments. **c** IP experiment assessing the interaction of ZEB1 truncations with ERα. Full-length ZEB1 and mutants were expressed with ERα in HEK293T cells. Predicted molecular weights are indicated (protein size markers are shown on the original uncropped blots available in the Source Data file). **d** Schematic overview of different ERα domains fused to the Gal4 DNA binding domain. **e** Gal4 luciferase reporter assays in HEK293T cells transiently coexpressing ZEB1 and Gal4-ERα domain fusion proteins as indicated. Ctl (control) is the plasmid pSCTEV gal93 for expression of the Gal4-DBD by itself. The luciferase activities in presence of indicated treatments are represented as fold activation over control vector values. For all luciferase reporter assays, statistical significance was determined with a two-way ANOVA (n = 3 biologically independent experiments). p-values are shown above the bars. All graphs with error bars represent the means ±SEM. Source data are provided as a Source Data file.

Supplementary Fig. 4

a

Motif	Factor	zScore	-log(pval)
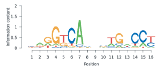	ESR1 (ERα)	-16.70	690.77
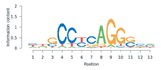	TFAP2C (AP2γ)	-11.23	664.47
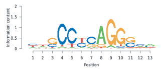	ZEB1	-10.23	473.75
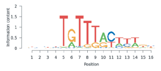	FOXA1	-8.36	379.81

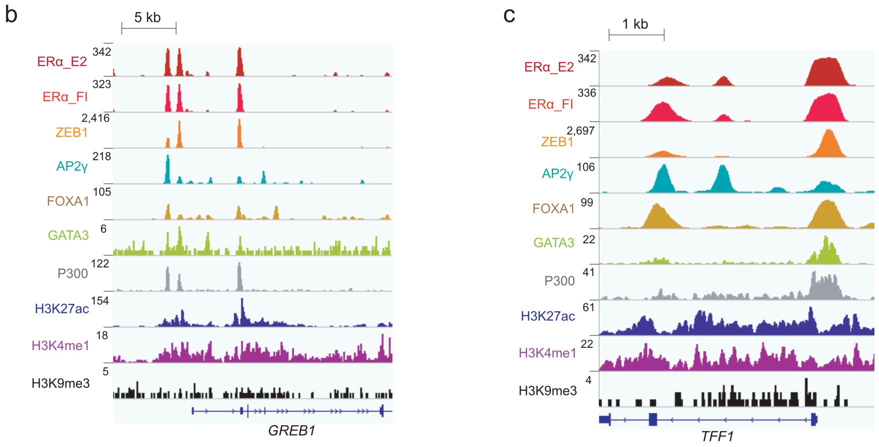

Supplementary Fig. 4 ZEB1 is a part of the ERα TF-complex during early EMT stages. a Motif enrichment analysis for the regions at the intersections of ZEB1 and E2- or FI-induced ERα binding sites (11,534 sites; see Venn diagram of Fig. 2j); motifs were identified by SeqPos analysis using known or *de novo* motif searches with the JASPAR motif matrix. ESR1 and TFAP2C stand for ERα and AP2γ, respectively. **b, c** Genome browser views, for the *GREB1* (**c**) and *TFF1* (**d**) genes as examples of the binding sites of ZEB1, ERα, AP2γ, FOXA1, GATA3, and P300, and the open chromatin histone marks H3K27ac, H3K4me1, and H3K9me3. Except for ERα and ZEB1, ChIP-seq data were from publicly available datasets: GSE21234 (TFAP2C), GSE25315 (FOXA1), GSE60270 (GATA3, P300, H3K27ac, H3K4me1, and H3K9me3).

Supplementary Fig. 5

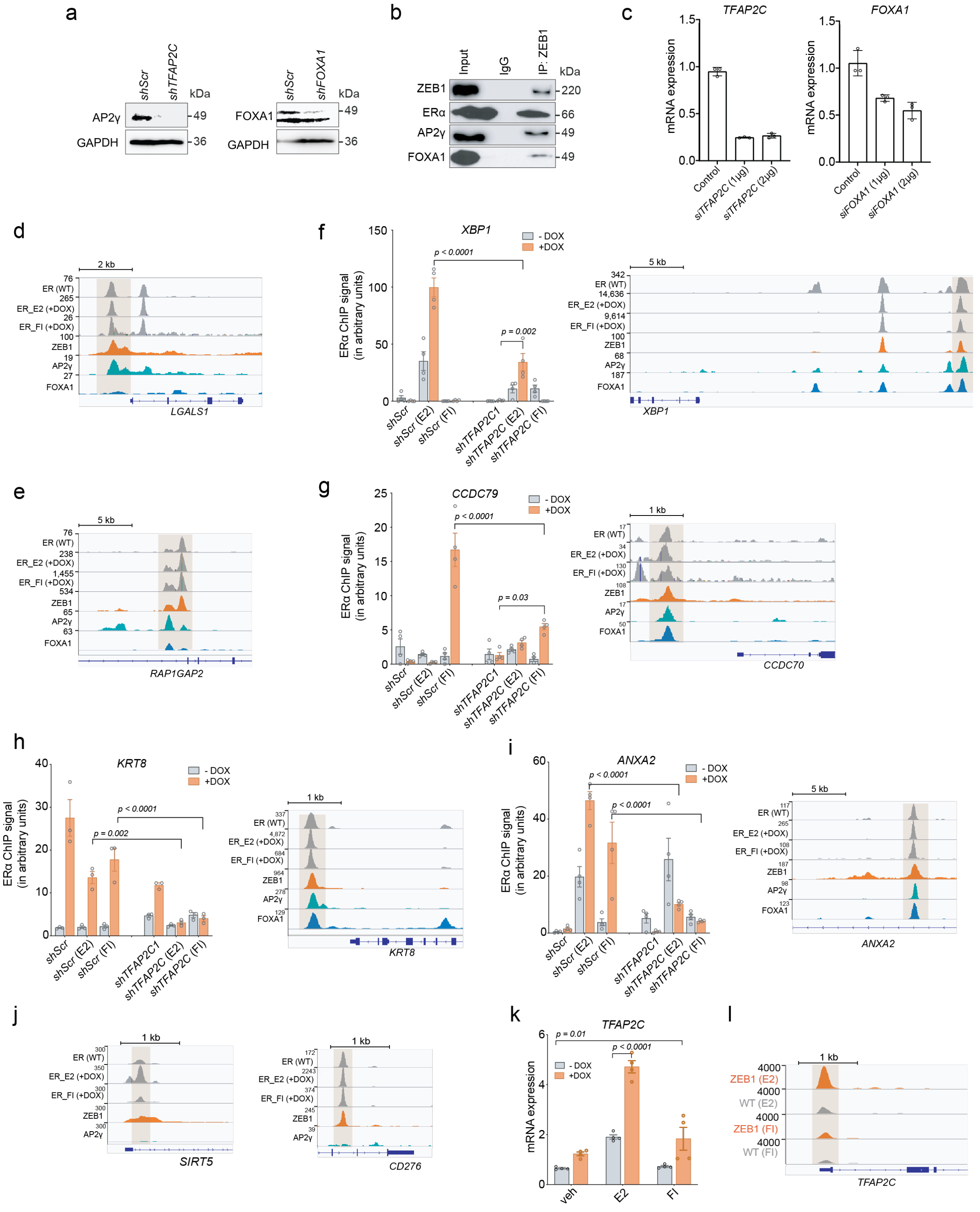

Supplementary Fig. 5 AP2γ determines the interaction between ZEB1 and ERα. **a** Immunoblots presenting the knockdown efficiencies in cells infected with lentiviral constructs for shRNAs targeting *TFAP2C* and *FOXA1* mRNAs. Scrambled shRNA (shScr) was used as negative control. **b** Immunoblots of ZEB1, ERα, AP2γ, and FOXA1 in ZEB1 IPs. An IP with an IgG antibody was used as a negative control. **c** RT-qPCR analyses in cells transiently transfected with a mixture of siRNAs targeting

TFAP2C (left panel) or *FOXA1* (right panel) in HEK293T cells. A universal negative control siRNA was used as "Control" (n = 3 biologically independent experiments). **d** and **e** Genome browser snapshots of the ERα, ZEB1, AP2γ, and FOXA1 ChIP-seq data at the *LGALS1* (**d**) and *RAP1GAP2* (**e**) genes. **f-i** ERα ChIP-qPCR for assessing the impact of a *TFAP2C* knockdown at candidate ERBSs (n = 4 biologically independent experiments, except for panel **h**, which is based on n = 3 biologically independent experiments); for **f-i** to the right of the bar graphs the corresponding genome browser views are shown. **j** Genome browser snapshots of the ERα, ZEB1, and AP2γ ChIP-seq data at the *SIRT5* and *CD276* genes. **k** *TFAP2C* mRNA expression levels were analyzed by RT-qPCR following 6 h of treatments as indicated (n = 4 biologically independent experiments). **l** Genome browser snapshot of the ERα ChIP-seq data at the *TFAP2C* gene. The sources for ChIP-seq datasets for factors other than ERα and ZEB1 are as indicated in the legend to Supplementary Fig. 4. veh, E2, and FI stand for vehicle, 17β-estradiol, and forskolin + IBMX, respectively. All error bars represent standard errors of the means (mean ± SEM). p-values are shown above the bars; statistical significance was determined with a two-way ANOVA. Source data are provided as a Source Data file.

Supplementary Fig. 6

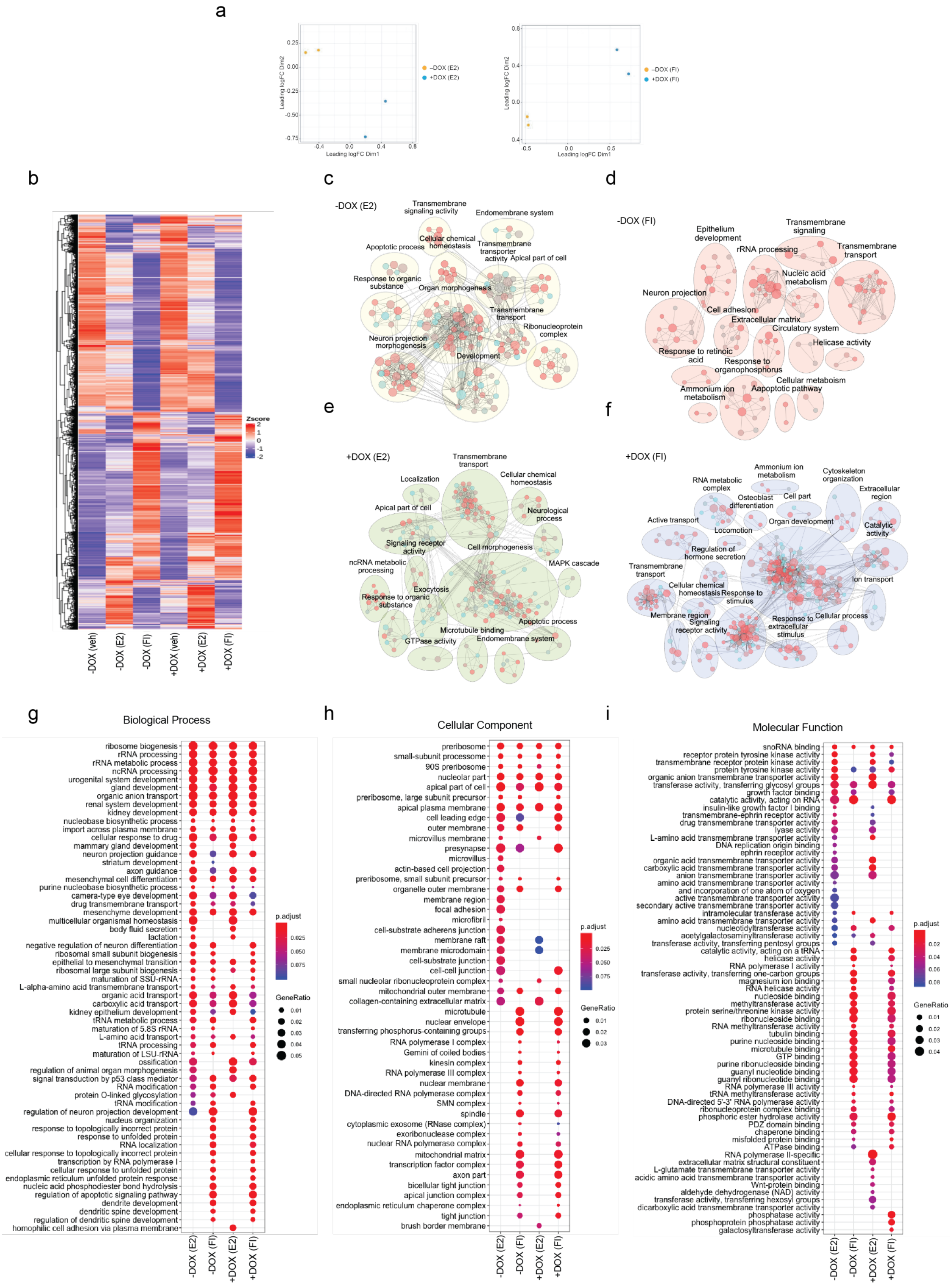

Supplementary Fig. 6 GO and GSEA analyses of the RNA-seq data. a Multi-dimensional scaling plot of RNA-seq data in two dimensions. Leading log fold-change (logFC) is the mean logFC between the most differentially expressed genes between each pair of samples. **b** Clustering heat map of the RNA-seq data (–DOX (vehicle), –DOX (E2), –DOX (FI), +DOX (vehicle), +DOX (E2), and +DOX (FI)), based on merged replicates from two independent biological replicates. **c-f** Cytoscape enrichment map visualization of a GSEA of the RNA-seq data comparing the functions of gene set clusters in each of the following groups: –DOX (E2) (**c**), +DOX (E2) (**d**), –DOX (FI) (**e**), and +DOX (FI). (**f**). Red node color indicates positive gene set enrichment and augmentation of the function, and blue nodes are indicative of suppression of that function. Node size represents the number of genes enriched in each function (FDR < 0.05). **g-i** Bubble plots of top GO terms compared in the four groups: –DOX (E2), –DOX (FI), +DOX (E2), and +DOX (FI). The GO classes were separated into three major categories: biological process (**g**), cellular component (**h**) and molecular function (**i**). veh, E2, and FI stand for vehicle, 17β-estradiol, and forskolin + IBMX, respectively. The ClusterProfiler package in R was used to classify gene sets with the GO terms "Biological Process", "Cellular Component", and "Molecular Function". p-values are shown by different colors and the sizes of the bubbles indicate the gene count of each pathway. Source data are provided in Supplementary Data 3-5.

Supplementary Fig. 7

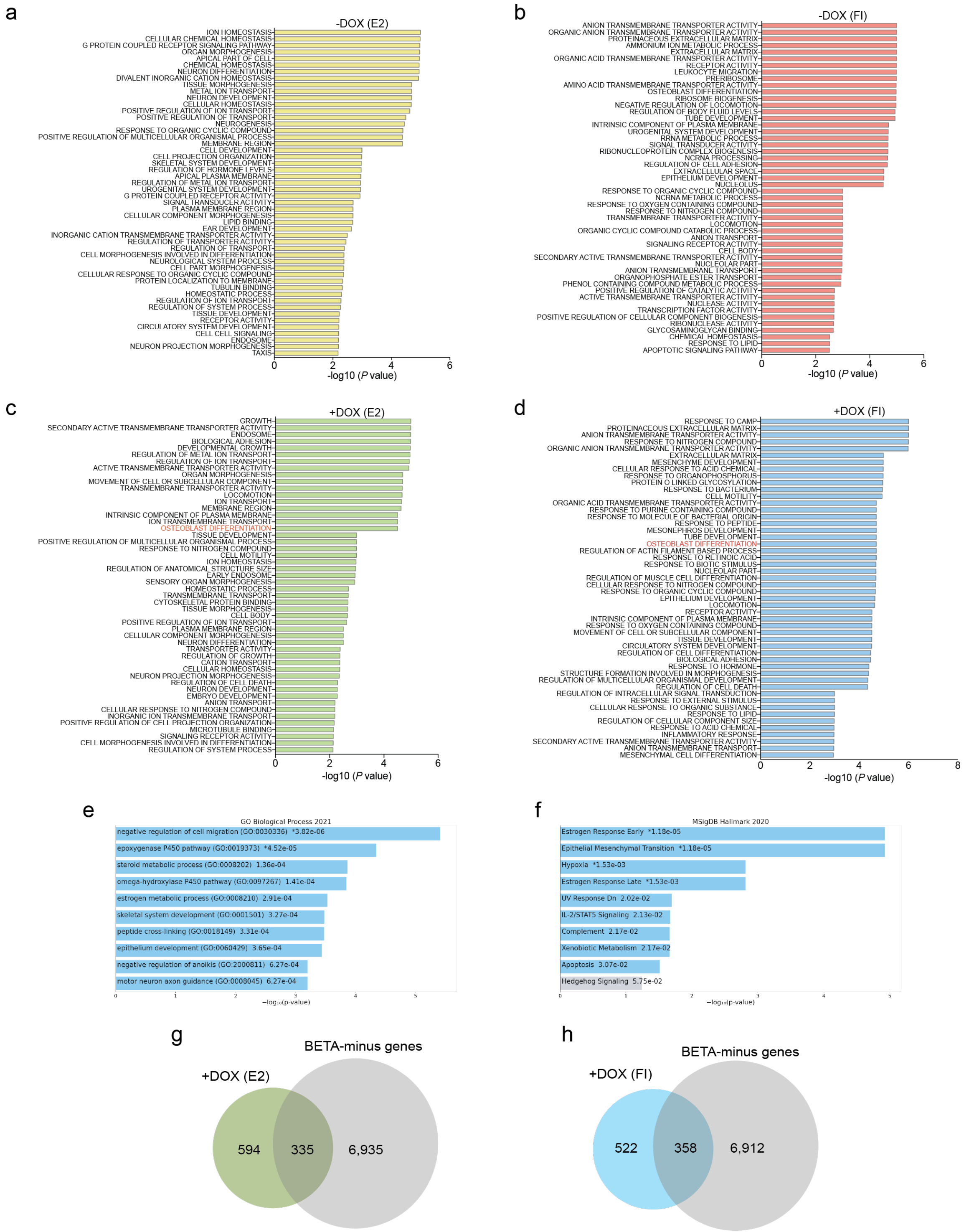

Supplementary Fig. 7 GO analysis of the RNA-seq data. a-d The top 50 GO terms generated from the RNA-seq data by GSEA of differentially expressed ERα target genes in MCF7-V-ZEB1 cells with (+DOX) or without (–DOX) ZEB1 and stimulated with E2 or FI. Bar plots show the -log10(p-value) of GO terms associated with genes whose expression significantly changes (-log10(p-value) > 1.5) with E2 and FI treatments with or without ZEB1 expression. E2 and FI stand for 17β-estradiol and

forskolin + IBMX, respectively. **e, f** The bar charts show the top 10 enriched GO terms in the indicated GO categories, with their corresponding p-values. Blue bars correspond to terms with p-values <0.05; an asterisk (*) indicates that the term also has a significant adjusted p-value (<0.05). Gene set analysis was performed with Enrichr (https://maayanlab.cloud/Enrichr/), based on the Kolmogorov-Smirnov test. **g, h** Venn diagrams showing the intersections between uniquely expressed genes unlocked by ZEB1 in MCF7-V-ZEB1 cells treated with E2 (**e**) or FI (**f**) and genes identified by BETA analysis of shared ZEB1 and ERα binding sites. Source data are provided as a Source Data file.

Supplementary Fig. 8

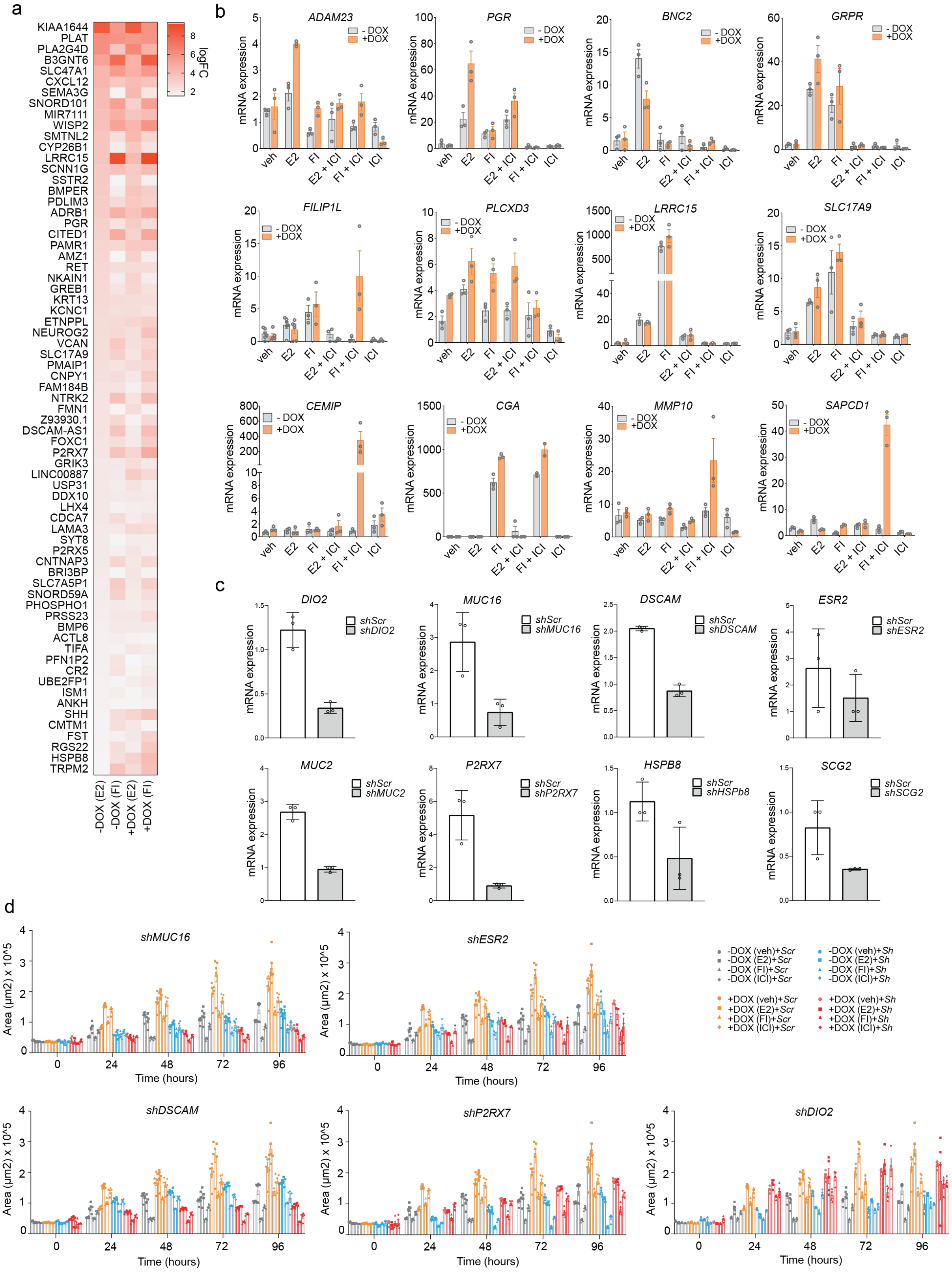

Supplementary Fig. 8 Expression and functional analyses of ERα target genes. **a** Heatmap showing the log2 fold changes (log2FC) of top differentially expressed genes, based on the RNA-seq analysis of MCF7-V-ZEB1 cells. **b** Bar graphs showing mRNA levels of selected genes, based on an RT-qPCR analysis (n = 3 biologically independent experiments). All error bars represent standard errors of the means (mean ± SEM). **c** Knockdown efficiencies of selected genes assessed by RT-qPCR. n = 3 biologically independent experiments. All error bars represent standard errors of the means (mean ± SEM). **d** Bar graphs representing the changes during 96 h in the invasion ability of spheroids with (+DOX) or without (–DOX) ZEB1 expression in cells infected with scrambled RNA (scrRNA) or shRNAs targeting the *MUC16, ESR2, DSCAM, P2RX7*, and *DIO2* mRNAs. veh, E2, FI, and ICI stand for vehicle, 17β-estradiol, forskolin + IBMX, and fulvestrant, respectively. Bars show means ± SEM, based on n = 3 biologically independent experiments, each with n = 2 replicate wells. Source data are provided as a Source Data file.

Supplementary Fig. 9

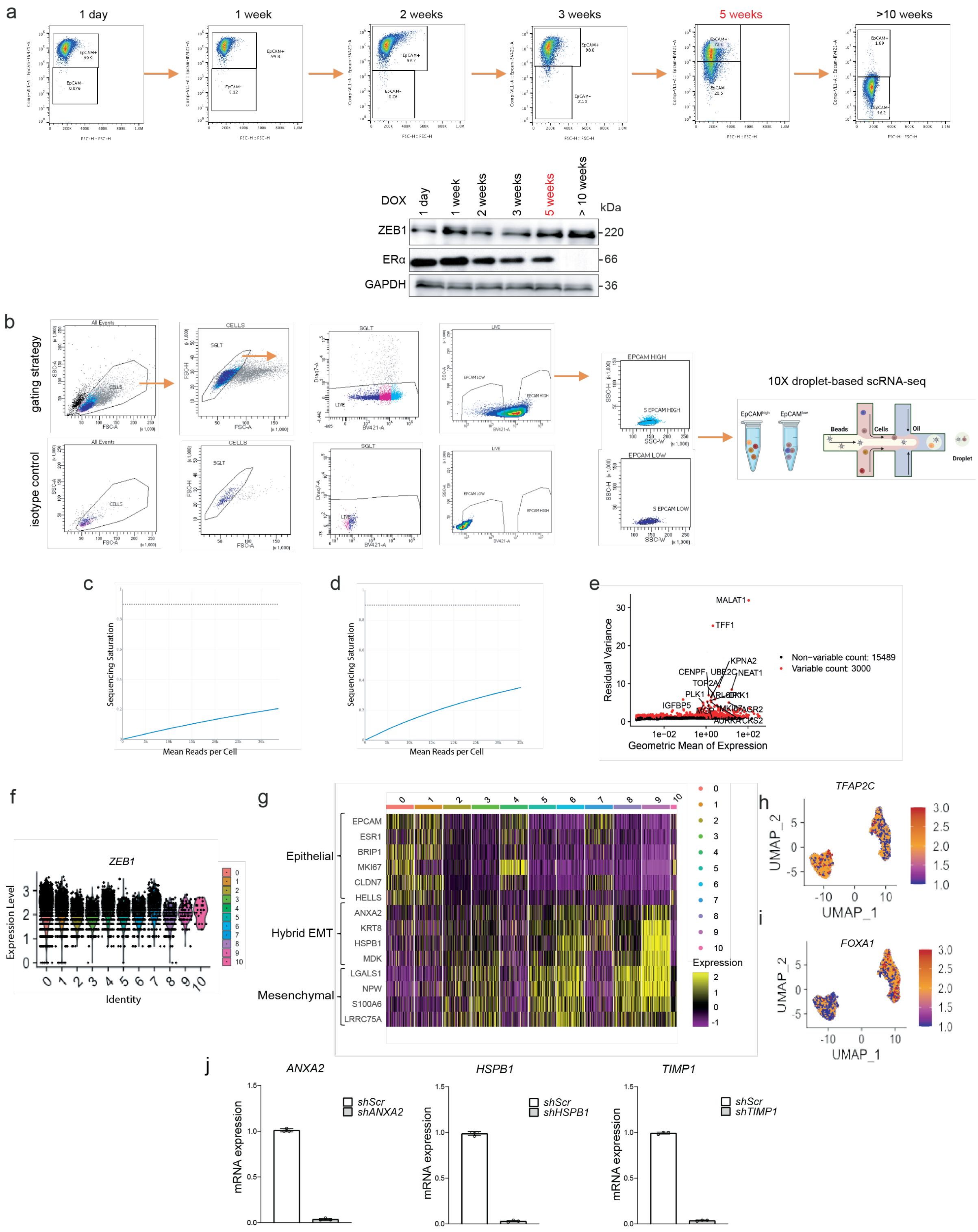

Supplementary Fig. 9 FACS analysis strategy and scRNA-seq. a Representative dot plots of EpCAM quantitation by FACS. Time-course of cells gated into EpCAM^{high} and EpCAM^{low} cell populations following DOX-induced ZEB1 expression in MCF7-V-ZEB1 cells. Immunoblots of ZEB1, ERα, and GAPDH corresponding to cell extracts corresponding to each time point shown in the upper panels (results are representative

of three independent experiments). Cells of the 5 weeks' time point were used for the scRNA-seq analysis. **b** Gating strategy for the FACS-sorting of EpCAM-stained cells after treatment with DOX for 5 weeks for the scRNA-seq analysis of $EpCAM^{high}$ and $EpCAM^{low}$ cell populations. An IgG1 isotype control was used to exclude non-specific antibody bindings. The scheme on the right was created with BioRender.com. **c, d** Sequencing Saturation plots of $EpCAM^{high}$ (**c**) and $EpCAM^{low}$ (**d**) cell populations. **e** Variable feature plot highlighting the 15 most highly variable genes from the normalized scRNA-seq data of the combined scRNA-seq datasets of the $EpCAM^{high}$ and $EpCAM^{low}$ cell populations. **f** Violin plot indicating the distribution of the ZEB1 mRNA in single cells across different clusters. **g** Heatmap representing the heterogenous expression of specified genes from the epithelial, hybrid EMT, and mesenchymal states distributed across the 11 clusters of the scRNA-seq. **h, i** Feature plots showing the expression of *TFAP2C* (**h**) and *FOXA1* (**i**) at the single-cell level. **j** Knockdown efficiencies for the *ANXA2*, *HSPB1*, and *TIMP1* mRNAs, assessed by RT-qPCR. n = 3 biologically independent experiments. All error bars represent standard errors of the means (mean ± SEM). Source data are provided as a Source Data file.

Supplementary Fig. 10

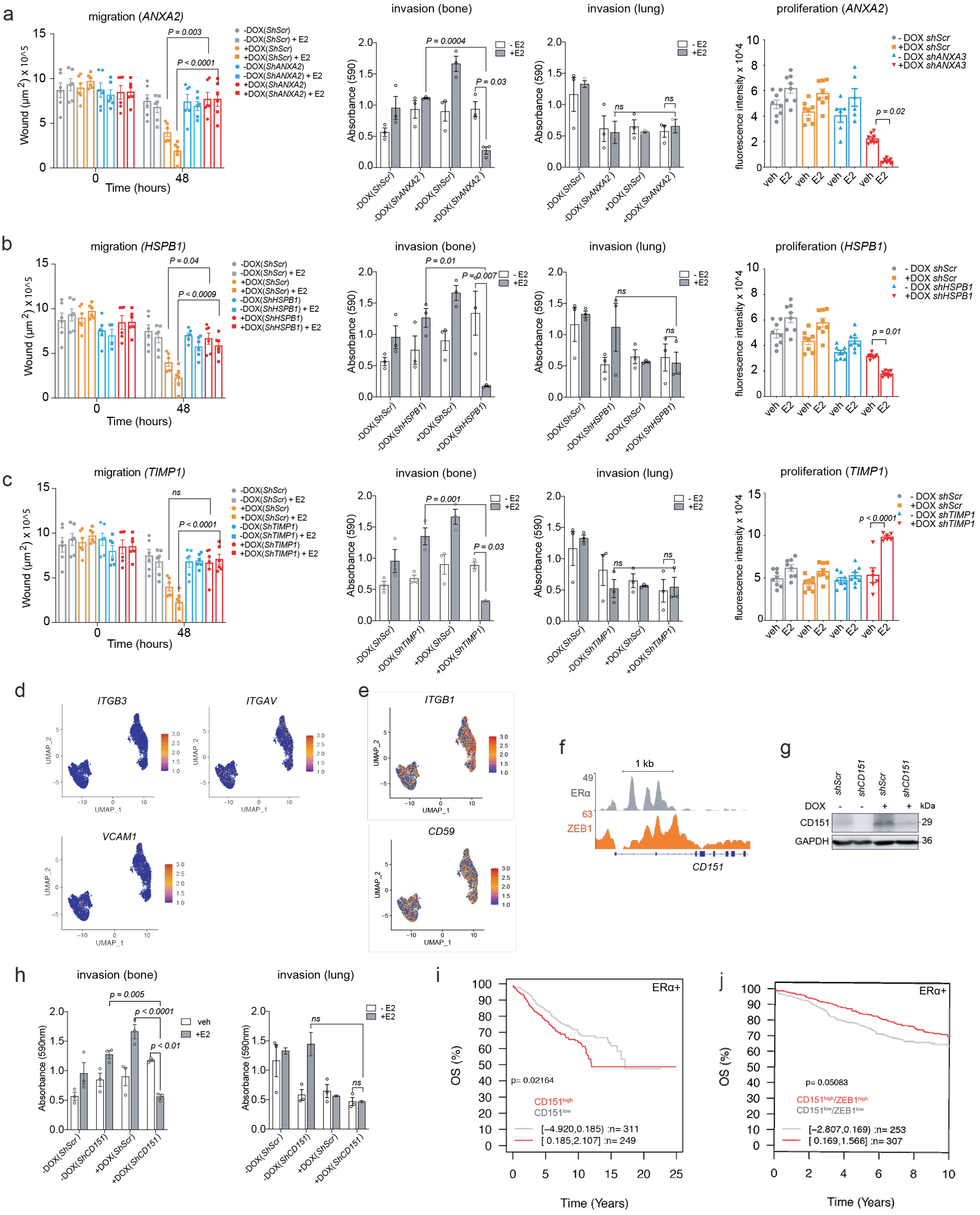

Supplementary Fig. 10 ZEB1-induced markers of EMT hybrid-state are involved in cell migration and invasion in an ERα-dependent manner. a-c Panels show different functional assays for the markers *ANXA2* (**a**), *HSPB1* (**b**), and *TIMP1* (**c**), which were identified in the scRNA-seq data experiment. All assays were performed with MCF7-V-ZEB1 cells infected with viruses for the expression of the indicated

shRNAs. The bar graphs in the leftmost panels show the quantification of migration in a wound healing assay with –DOX and +DOX cells treated with veh or E2 for 48 h; the Y-axis shows the remaining wound area into which cells have *not* yet migrated. Middle panels illustrate the quantification of invasion of infected cells towards bone or lung tissues in presence of veh or E2 for 48 h. Bar graphs in the rightmost panels show the viability of infected –DOX and +DOX cells exposed to veh or E2 for 72 h (n = 3 independent experiments each including n = 2 technical replicates for migration assays; n = 3 independent experiments for invasion assays; n = 4 independent experiments each including n = 2 technical replicates for proliferation assays; means ± SEM). p-values are shown above the bars; statistical significance was determined with two-way ANOVA and error bars indicate mean ± SEM. Source data are provided as a Source Data file. **d** Feature plots showing the distribution of single cell transcripts for the *ITGB3* (CD61), *ITGAV* (CD51) and *VCAM1* (CD106) genes. **e** Feature plots presenting the distribution of *ITGB1* (CD29) and *CD59* gene expression levels from single cells. **f** Genome browser view of ERα and ZEB1 binding associated with the *CD151* gene; extracted from the respective ChIP-seq data. **g** Control immunoblots showing the efficiency of knockdown of CD151 in cells targeted with a pool of two different shRNAs (results are representative of n = 2 independent experiments). **h** Quantification of a transwell assay for invasion of MCF7-V-ZEB1 cells, infected with lentiviruses for expression of CD151 shRNAs or control shRNA, towards bone or lung tissues after 48 h of treatment with veh or E2. Error bars show means ± SEM, n = 3 biologically independent experiments. p-values are shown above the bars; statistical significance was determined with two-way ANOVA. Source data are provided as a Source Data file. **i, j** Kaplan-Meier plots for overall survival (OS) analyses of ERα^+ breast cancer patients classified as tumors expressing high or low levels of CD151 (**i**) and high or low levels of both ZEB1 and CD151 (**j**).

Supplementary Fig. 11

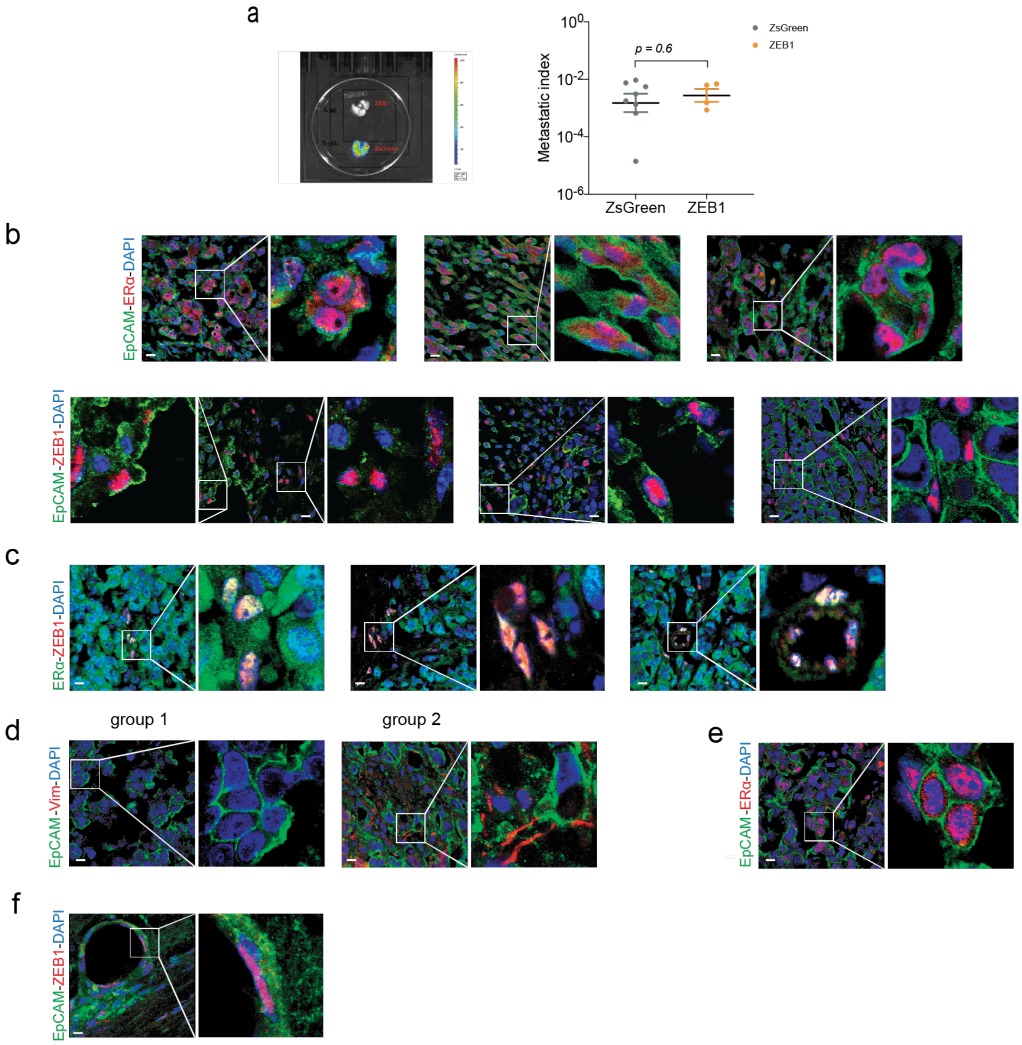

Supplementary Fig. 11 ZEB1 induces metastasis *in vivo*. a Left panel is the BLI image showing metastatic lesions in lungs of ZsGreen- or ZEB1-expressing wild-type MCF7 cells. The panel on the right is the bar graph of the metastatic indices (ratio of average radiance of metastases over average radiance of corresponding primary tumor; see Methods for definition and mode of calculation of average radiance) for lungs; note that the data are shown after log10 transformation; an unpaired and two-tailed Student's t-test was used for the statistical analysis (n = 8 independent animals for ZsGreen and n = 4 for ZEB1 cells, means ± SEM). **b** IF images of EpCAM, ZEB1, and ERα and DAPI staining of resected tumors formed by MCF7-ZEB1 cells (+DOX) following their orthotopic injection into mouse mammary fat pads. **c** IF images of ERα and ZEB1 from MCF7-ZEB1 tumors. **d, e** IF images of EpCAM with vimentin (**d**) and with ERα (**e**) from resected primary tumors formed by MCF7-ZsGreen control cells. **f** IF staining of EpCAM and ZEB1 in metastatic lesions of femur bones of mice with MCF7-ZEB1 tumors. Images are from n ≥ 6 independent experiments. Scale bars = 10 µm.

Supplementary Tables

Supplementary Table 1. Details of the shRNA target sequences used for the knockdown experiments.

shRNA	Target sequence 5'-3'	Reference / TRCN*/manual design**
sh1*ANXA2*	CGGGATGCTTTGAACATTGAA	TRCN0000056145
sh2*ANXA2*	TGAGGGTGACGTTAGCATTAC	TRCN0000296322
sh1*CD151*	ACCTGCTGTTTACCTACAATT	TRCN0000057646
sh2*CD151*	GCAGTCACCACCACCCGAAAT	TRCN0000381613
sh*DIO2*	GTATTGCCTTGGCTCTATTTG	TRCN0000294313
sh1*DSCAM*	AAAGAGTTTAGCTGAAATGCT	ref. 9
sh2*DSCAM*	CCTCCCGAAATTGAGATCAAA	Designed manually
sh1*ESR2*	CGCCAGTTATCACATCTGTAT	ref. 10
sh2*ESR2*	AGGCCATGATCCTGCTCAATT	ref. 10
sh1*FOXA1*	GCGAAGTTTAATGATCCACAA	TRCN0000014878
sh2*FOXA1*	GAACACCTACATGACCATGAA	ref. 11
sh1*HSPB1*	CCGATGAGACTGCCGCCAAGT	TRCN0000008753
sh2*HSPB1*	GATCACCATCCCAGTCACCTT	ref. 12
sh1*MUC2*	CGGAGTTTACATCGACAACTA	TRCN0000073548
sh2*MUC2*	CGACTACAAGATACGTGTCAA	TRCN0000073552
sh*MUC16*	GCCAAGACTTTGGCTTCAGAA	TRCN0000180917
sh1*P2RX7*	CCTGGCTACAACTTCAGATAC	ref. 13
sh2*P2RX7*	CCTGGCTACAACTTCAGATAC	Designed manually
sh1*TFAP2C*	GCTGAGCTATCTCCTAACTTT	TRCN0000019744
sh2*TFAP2C*	CCTCAGCTCTACGTCTAAATA	TRCN0000019746
sh1*TIMP1*	ACAGTGTTTCCCTGTTTAT	Designed manually
sh2*TIMP1*	CCACCTTATACCAGCGTTATA	TRCN0000080039
shScr	CCTAAGGTTAAGTCGCCCTCG	ref. 14

* TRCN: The RNA consortium number (https://www.broadinstitute.org/rnai/trc)
** shRNAs were designed manually with an online tool at http://sirna.wi.mit.edu using the gene of interest accession number.

Supplementary Table 2. Primer sequences used in the ChIP and re-ChIP qPCR experiments.

ChIP and re-ChIP	Forward primer 5'-3'	Reverse primer 5'-3'
ANXA2	GAGAGAAAGGCCTCAGCAACT	CTTCTGTGACTCACGTAGCCC
ANXA3	CTGACGGGTGGCTTGGG	ACTGAGGTCCCTCCAGCTC
c-MYC intron	GCCAGTCCAACCGGCTTATG	GGTTCTCCCAAGCAGGAGCA
CCDC79	TGACCTGTTTGGCAGTTCAC	ACAAATGCCCTGAGGATAGG
CD276	TCTGGGCATCAGTTTCCCATC	TGTCCTTTGCAAGTACCTCCC
CDH1 +8 kb	TCACCCTGCCTGCTTCTGTGT	ACCCTGAGCACCCTGCACA
CDH1 +0.5 kb	GGCCGGCAGGTGAACCCTCA	GGGCTGGAGTCTGAACTGA
CEP89	ACTCCCTGATGCATTGAGCA	GCCCCTGGACATTTGACTGA
GREB1 -20 kb	ACACCCCACCAGCTAGTACA	ACAGCCGTGTGAGACAAGAG
GREB1 -3 kb	ACTGGAGGTCAGCTCAGTCA	GCGTCAAGCAACTACACTCC
GREB1 +5 kb	GTACTGTGGCTCCAGTCCAAGT	CAAATGCCACCGTTTCGTGTCT
KRT8	CAGAACCTGGAAGGAGCAAG	GCCCCACCAGATAGTCTCCT
LAMC2 -96 bp[15]	TGTGCTCTGTGTGTTTGTCTGCC	TGTGAATAAGGAGGACCCAGCC
LGALS1	GGCCATCTCCTAAGCTTGAC	CCTCGGGAAGGCTAAAGAAA
MYC TSS	TGAGTATAAAAGCCGGTTTTC	TCCCTTCCCAGGACGCCCGCA
RAP1GAP2	GAGAAAGCCCGTATTGACCA	GCCTGTGAAGTGGAGCAGAT
RARA +30 kb	TTGTTCTGCTACAGCCAGGGT	CTGACCCGTAGTGCACACAGAA T
SIRT5	GTAATCCCACCCGAGCAGGT	GCAGCTGCAATGACCAGGGT
SLC25A24	AGTGAGATTGAGTGACTTTCCC A	AGGTGGTTGGAACCAGACAAA
TBX2	CGGAGCTGGGTCGGAGG	GCAAAGCATTGACACGACCC
TFAP2C	GGAGCGGTCTTGACACTCG	CCCCCAAGCGGTAAATCCAA
TFF1 -9 kb	AGCAAGACCAGGGTTGCAT	ACGAGCAGATGGAGGTGGGT
TFF1 +0.5 kb	CACCCCGTGAGCCACTGT	CTGCAGAAGTGATTCATAGTGAG
TFF1 +2 kb	GCAATGGGTTTCCACCTCCT	CACCAGGTGAAGATGGAGCC
XBP1 -9 kb	ATACTTGGCAGCCTGTGACC	GGTCCACAAAGCAGGAAAAA
XBP1 up	GCAAACAATAGCCCAGAAGC	GTCCAAGGGCACATTCTCAT

Supplementary Table 3. Primer sequences used for RT-qPCR.

mRNA	Forward primer 5'-3'	Reverse primer 5'-3'
ADAM23	GACCCAAGGGTCCTAGTGC	GATGGGGCCTTGCTGAGTAG
ADRB1	CCGGGAACAGGAACACAC	GAAAGCAAAAGGAAATATGTC
ANXA2	TCGGACACATCTGGTGACTTCC	CCTCTTCACTCCAGCGTCATAG
BMPER	TGTGTTTGAGGGTGTGCAGT	ATTGTGTCCTGCCTCCAGTG
BNC2	AAATCAGAGGACAGGCTTAGTGA	TTGAGATGTATCAACCCCACAAC
CCDC110	CTGTCCTTCCTCGACCAAACA	AACACACTGCTCAGTAACGGA
CEMIP	ACCGAGCACATTCCAACTACCG	GGCAGAGATGATTGAGAGGAACG
CGA	TGTGCAGGATTGCCCAGAAT	CTGAAGTATTGGGGCACCCG
CXCL12	AACACTCCAAACTGTGCCCT	AGTGGGTCTAGCGGAAAGTC
DIO2	TCGATGCCTACAAACAGGTGAA	CTGGGTACCATTGCCACTGT
DSCAM	ATCGGCCAGGTGGTCCAG	TCCGGCTTTTCTGAGGTTCC
ESR2	AGAGTCCCTGGTGTGAAGCAA	GACAGCGCAGAAGTGAGCATC
FILIP1L	GAACTGCAGGCTCGAGATGA	TGAGCTTCCAGCAAAGCCAG
GAPDH	GCACAAGAGGAAGAGAGAGACC	AGGGGAGATTCAGTGTGGTG
GREB1	GGCAGGACCAGCTTCTGA	CTGTTCCCACCACCTTGG
GRPR	CGCTCTCGGCAGACAGATAC	GGTCTGGTTGGTGCTTTCCT
HSPB1	CGCGGAAATACACGCTGC	GTGATCTCGTTGGACTGCGT
HSPB8	GTGTGTGAATGTGCACAGCTT	TCATGTTTGCCAGACACCTCC
MMP10	GACAGAAGATGCATCAGGCAC	AGCTTCAGTGTTGGCTGAGT
MUC16	CCTTAACGGTTACAATGAACCTGG	GTGTGAGGGTCTTCAGGTGG
MUC2	CTGCTATGTCGAGGACACCC	GAGTTGGTACACACGCAGGA
MYC	CACCTTGTAGCACGTCCTG	GACTCCCCAAGATGTGGTGG
P2RX7	ACGTTTGCTTTGCTCTGGTG	ACCTTGGTGTGCACAGAACT
PGR	AGCCAGAGCCCACAATACAG	AGTTGTGCTGCCCTTCCATT
PLCXD3	CTCGTCTCAGGGGAAAAACGA	ATCATGAGACCCTGGAATGGC
SAPCD1	TTTACACTCAGAGCCTGGTCGC	CTAGCCTTGTTCAGTGGACTTGG
SCG2	CGGAGAACGGGGAGGAATATG	GCCATGTTTGAAAGATTTCCTCTT
SLC17A9	TCTCCAGCGTCTTTGCTCTG	GCCACTGTGGTTGAAGGTCT
TIMP1	TGTGAGGAATGCACAGTGTT	TCCACAAGCAATGAGTGCCA
XBP1	CCCTCCAGAACATCTCCCCAT	ACATGACTGGGTCCAAGTTGT

Supplementary Table 4. Primer sequences used for the Gibson assemblies related to the generation of plasmids for expression of ZEB1 truncation mutants.

ZEB1 mutants	Primers
Insert 1 ΔZF1	F: 5' GAACCGTCAGATCGCACCGGCCGGTGAGGACCATGGCATATC 3'<br>R: 5' TCAAGCTAATCAACTGGGAAAATGCATCTGGTG 3'
Insert 2 ΔZF1	F: 5' TTCCCAGTTGATTAGCTTGATGCCTGTGAATGG 3'<br>R: 5' TCAGGATCCGGTTACCTCGATCGAGCCTTCTGTTGGGTTAACC 3'
Insert 1 ΔZF2	F: 5' GAACCGTCAGATCGCACCGGCCGGTGAGGACCATGGCATATC 3'<br>R: 5' AGGAGTAGCGCATTCCATTCTCTGTCTTCCGAGT 3'
Insert 2 ΔZF2	F: 5' GAATGGAATGCGCTACTCCTACTGCAAGAGAGG 3'<br>R: 5' TCAGGATCCGGTTACCTCGATCGAGCCTTCTGTTGGGTTAACC 3'
Insert 1 ΔZF1/2	F: 5' GAACCGTCAGATCGCACCGGCCGGTGAGGACCATGGCATATC 3'<br>R: 5' TCAAGCTAATCAACTGGGAAAATGCATCTGGTG 3'
Insert 2 ΔZF1/2	F: 5' TTCCCAGTTGATTAGCTTGATGCCTGTGAATGG 3'<br>R: 5' AGGAGTAGCGCATTCCATTCTCTGTCTTCCGAGT 3'
Insert 3 ΔZF1/2	F: 5' GAATGGAATGCGCTACTCCTACTGCAAGAGAGG 3'<br>R: 5' TCAGGATCCGGTTACCTCGATCGAGCCTTCTGTTGGGTTAACC 3'
Insert 1 ΔHD	F: 5' GAACCGTCAGATCGCACCGGTGAGGACCATGGCATATCC 3'<br>R: 5' GGTCAGGAGATCCGTTTCCAGCTGATGAAGC 3'
Insert 2 ΔHD	F: 5' TGGAAACGGATCTCCTGACCCCCCTTCTCC 3'<br>R: 5' TCAGGATCCGGTTACCTCGAGCCTTCTGTTGGGTTAACCTGAAG 3'

Supplementary References

1. Zhang, P., Sun, Y. & Ma, L. ZEB1: at the crossroads of epithelial-mesenchymal transition, metastasis and therapy resistance. *Cell Cycle* **14**, 481-487 (2015).
2. Vandewalle, C., Van Roy, F. & Berx, G. The role of the ZEB family of transcription factors in development and disease. *Cell. Mol. Life Sci.* **66**, 773-787 (2009).
3. Koide, A. et al. Identification of regions within the F domain of the human estrogen receptor α that are important for modulating transactivation and protein-protein interactions. *Mol. Endocrinol.* **21**, 829-842 (2007).
4. Seipel, K., Georgiev, O. & Schaffner, W. Different activation domains stimulate transcription from remote ('enhancer') and proximal ('promoter') positions. *EMBO J.* **11**, 4961-4968 (1992).
5. Gburcik, V., Bot, N., Maggiolini, M. & Picard, D. SPBP is a phosphoserine-specific repressor of estrogen receptor α. *Mol. Cell. Biol.* **25**, 3421-3430 (2005).
6. Maggiolini, M. et al. Estrogen receptor α mediates the proliferative but not the cytotoxic dose-dependent effects of two major phytoestrogens on human breast cancer cells. *Mol. Pharmacol.* **60**, 595-602 (2001).
7. Carascossa, S., Dudek, P., Cenni, B., Briand, P. A. & Picard, D. CARM1 mediates the ligand-independent and tamoxifen-resistant activation of the estrogen receptor α by cAMP. *Genes Dev.* **24**, 708-719 (2010).
8. Webb, P. et al. Estrogen receptor activation function 1 works by binding p160 coactivator proteins. *Mol. Endocrinol.* **12**, 1605-1618 (1998).
9. Purohit, A. A. et al. Down syndrome cell adhesion molecule (DSCAM) associates with Uncoordinated-5C (UNC5C) in Netrin-1-mediated growth cone collapse. *J. Biol. Chem.* **287**, 27126-27138 (2012).
10. Dondi, D. et al. Estrogen receptor β and the progression of prostate cancer: role of 5α-androstane-3β,17β-diol. *Endocr. Relat. Cancer* **17**, 731-742 (2010).
11. Ni, M. et al. Amplitude modulation of androgen signaling by c-MYC. *Genes Dev.* **27**, 734-748 (2013).
12. Sevin, M. et al. HSP27 is a partner of JAK2-STAT5 and a potential therapeutic target in myelofibrosis. *Nat. Commun.* **9**, 1431 (2018).
13. Gui, Y. et al. Uridine adenosine tetraphosphate induces contraction of airway smooth muscle. *Am. J. Physiol. Lung Cell. Mol. Physiol.* **301**, L789-794 (2011).
14. Sarbassov, D. D., Guertin, D. A., Ali, S. M. & Sabatini, D. M. Phosphorylation and regulation of Akt/PKB by the Rictor-mTOR complex. *Science* **307**, 1098-1101 (2005).
15. Sanchez-Tillo, E. et al. β-catenin/TCF4 complex induces the epithelial-to-mesenchymal transition (EMT)-activator ZEB1 to regulate tumor invasiveness. *Proc. Natl. Acad. Sci. USA* **108**, 19204-19209 (2011).
