# Supplementary material for: Cooperative interaction between ERα and the EMT-inducer ZEB1 reprograms breast cancer cells for bone metastasis
Source: Nat Commun. 2022 Apr 19;13:2104. doi: 10.1038/s41467-022-29723-5 (PMC9018728; doi:10.1038/s41467-022-29723-5)
Supplement: Supplementary file 3 — Description of Additional Supplementary Information [file 41467_2022_29723_MOESM3_ESM.pdf]

Description of Additional Supplementary Information

The Supplementary Information file includes

- Supplementary Note 1
- Supplementary Methods
- 11 Supplementary Figures with legends
- 4 Supplementary Tables
- Supplementary References
