# Supplementary material for: Cooperative interaction between ERα and the EMT-inducer ZEB1 reprograms breast cancer cells for bone metastasis
Source: Nat Commun. 2022 Apr 19;13:2104. doi: 10.1038/s41467-022-29723-5 (PMC9018728; doi:10.1038/s41467-022-29723-5)
Supplement: Supplementary file 10 — Reporting Summary [file 41467_2022_29723_MOESM10_ESM.pdf]

nature portfolio

Corresponding author(s): Didier Picard

Last updated by author(s): Mar 15, 2022

Reporting Summary

Nature Portfolio wishes to improve the reproducibility of the work that we publish. This form provides structure for consistency and transparency in reporting. For further information on Nature Portfolio policies, see our Editorial Policies and the Editorial Policy Checklist.

Statistics

For all statistical analyses, confirm that the following items are present in the figure legend, table legend, main text, or Methods section.

n/a	Confirmed	
☐	☒	The exact sample size (*n*) for each experimental group/condition, given as a discrete number and unit of measurement
☐	☒	A statement on whether measurements were taken from distinct samples or whether the same sample was measured repeatedly
☐	☒	The statistical test(s) used AND whether they are one- or two-sided<br>*Only common tests should be described solely by name; describe more complex techniques in the Methods section.*
☒	☐	A description of all covariates tested
☐	☒	A description of any assumptions or corrections, such as tests of normality and adjustment for multiple comparisons
☐	☒	A full description of the statistical parameters including central tendency (e.g. means) or other basic estimates (e.g. regression coefficient) AND variation (e.g. standard deviation) or associated estimates of uncertainty (e.g. confidence intervals)
☐	☒	For null hypothesis testing, the test statistic (e.g. *F*, *t*, *r*) with confidence intervals, effect sizes, degrees of freedom and *P* value noted<br>*Give P values as exact values whenever suitable.*
☒	☐	For Bayesian analysis, information on the choice of priors and Markov chain Monte Carlo settings
☒	☐	For hierarchical and complex designs, identification of the appropriate level for tests and full reporting of outcomes
☒	☐	Estimates of effect sizes (e.g. Cohen's *d*, Pearson's *r*), indicating how they were calculated

Our web collection on statistics for biologists contains articles on many of the points above.

Software and code

Policy information about availability of computer code

Data collection	*Provide a description of all commercial, open source and custom code used to collect the data in this study, specifying the version used OR state that no software was used.*

Data analysis

- FASTQ reads were aligned to the human genome hg19 using BWA-MEM (Version 0.7.17).
- For the ChIP-seq, peak calling was performed by the MACS2 tool (Version 2.1.0) of the Galaxy tool suite (https://usegalaxy.org). Motif analysis was performed with the SeqPos tool using the JASPAR motif matrix. Aggregation plots were generated using the cistrome platform (http://cistrome.org/ap/root).
- FeatureCounts (version 2.0.0) was then used to count reads per binding regions.
- The samtools program (version 1.10-3) from the hg19 genome was used for differential binding analysis.
- FIMO (version 5.0.5) using the HOCOMOCO v10 were used to identify specific binding motifs.
- The Integrative Genomics Viewer (IGV version 2.8.0) was used to browse and illustrate the binding sites.
- For RNA-seq analysis, sequences were mapped with the STAR (Version 2.7.0) and count tables were produced with the featureCounts function (Version 2.0.0) in R (version 3.6.2).
- For RNA-seq, "Gene set enrichment analysis" was carried out with GSEA (v4.1.0, Broad Institute) with the Gene ontology gene sets of the MSigDB collection. Enrichment Maps were generated by Cytoscape. The Integrative Genomics Viewer (IGV version 2.8.0) was used to browse and illustrate the binding sites.
- For the scRNA-seq, the Chromium Next GEM Single Cell 3' v3.1 workflow was followed for the library preparation and sequencing was performed with the Chromium Controller system from 10x Genomics.
- General analysis of count tables from all sequencing data sets were done with R (version 3.6.2).
- GSEA (v4.1.0) software was used for gene ontology analysis.
- Cytoscape version 3.8.2 was used to visualise GSEA results and enrichment maps.
- ImageJ (version 1.53c) software was used to analyze images.
- The Imaris 9.6 software was used to build the 2D and 3D images.

For manuscripts utilizing custom algorithms or software that are central to the research but not yet described in published literature, software must be made available to editors and reviewers. We strongly encourage code deposition in a community repository (e.g. GitHub). See the Nature Portfolio guidelines for submitting code & software for further information.

Data

Policy information about availability of data

All manuscripts must include a data availability statement. This statement should provide the following information, where applicable:

- Accession codes, unique identifiers, or web links for publicly available datasets
- A description of any restrictions on data availability
- For clinical datasets or third party data, please ensure that the statement adheres to our policy

- The data sets generated and/or analysed during the current study are publicly available in the GEO repository: Accession code GSE173562 at https://www.ncbi.nlm.nih.gov/geo/query/acc.cgi?acc=GSE173562
- The following publicly available ChIP-seq GEO datasets were used for comparisons: GSE109103 (ERalpha), GSE21234 (TFAP2C), GSE25315 (FOXA1), and GSE60270 (GATA3, P300, H3K27ac, H3K4me1, and H3K9me3).
- For scRNA-seq, the reference genome GRCh38-2020-A was downloaded from the 10X Genomics website.
- The Gene expression-based Outcome for Breast cancer Online (GOBO) tool is available at http://co.bmc.lu.se/gobo/
- Gene set analysis was performed with Enrichr at https://maayanlab.cloud/Enrichr/
- The Genomic Regions Enrichment of Annotations Tool (GREAT) is available at http://great.stanford.edu/public/html/

Field-specific reporting

Please select the one below that is the best fit for your research. If you are not sure, read the appropriate sections before making your selection.

☒ Life sciences ☐ Behavioural & social sciences ☐ Ecological, evolutionary & environmental sciences

For a reference copy of the document with all sections, see nature.com/documents/nr-reporting-summary-flat.pdf

Life sciences study design

All studies must disclose on these points even when the disclosure is negative.

Sample size

No sample size was pre determined. Based on the consistency of quantification differences between groups and replicates, sample sizes were determined to be adequate in an experimental setting. Therefore, three or more independent biological replicates were used to perform statistical analyses where applicable. For animal experiments, all mice were randomized before mouse experiments, blindly selected before injection and sample size was chosen based on power analysis.

Data exclusions

No data were excluded from the analysis.

Replication

Experiments in the article were reliably reproduced based on successful attempts to replicate experiments. The number of replicates are mentioned in the figure legends or in other parts of the text where applicable.

Randomization

No formal randomization technique was used, and samples were distributed based on different experimental conditions or treatments, and each group was identified appropriately during the study.

Blinding

Investigators were not blinded to group allocation during data collection and/or analysis. We used appropriate statistical tests to confirm significant differences; therefore, blinding was not applicable.

Behavioural & social sciences study design

All studies must disclose on these points even when the disclosure is negative.

Study description	*Briefly describe the study type including whether data are quantitative, qualitative, or mixed-methods (e.g. qualitative cross-sectional, quantitative experimental, mixed-methods case study).*
Research sample	*State the research sample (e.g. Harvard university undergraduates, villagers in rural India) and provide relevant demographic information (e.g. age, sex) and indicate whether the sample is representative. Provide a rationale for the study sample chosen. For studies involving existing datasets, please describe the dataset and source.*
Sampling strategy	*Describe the sampling procedure (e.g. random, snowball, stratified, convenience). Describe the statistical methods that were used to predetermine sample size OR if no sample-size calculation was performed, describe how sample sizes were chosen and provide a rationale for why these sample sizes are sufficient. For qualitative data, please indicate whether data saturation was considered, and what criteria were used to decide that no further sampling was needed.*
Data collection	*Provide details about the data collection procedure, including the instruments or devices used to record the data (e.g. pen and paper, computer, eye tracker, video or audio equipment) whether anyone was present besides the participant(s) and the researcher, and whether the researcher was blind to experimental condition and/or the study hypothesis during data collection.*
Timing	*Indicate the start and stop dates of data collection. If there is a gap between collection periods, state the dates for each sample cohort.*
Data exclusions	*If no data were excluded from the analyses, state so OR if data were excluded, provide the exact number of exclusions and the rationale behind them, indicating whether exclusion criteria were pre-established.*
Non-participation	*State how many participants dropped out/declined participation and the reason(s) given OR provide response rate OR state that no participants dropped out/declined participation.*
Randomization	*If participants were not allocated into experimental groups, state so OR describe how participants were allocated to groups, and if allocation was not random, describe how covariates were controlled.*

Ecological, evolutionary & environmental sciences study design

All studies must disclose on these points even when the disclosure is negative.

Study description	*Briefly describe the study. For quantitative data include treatment factors and interactions, design structure (e.g. factorial, nested, hierarchical), nature and number of experimental units and replicates.*
Research sample	*Describe the research sample (e.g. a group of tagged Passer domesticus, all Stenocereus thurberi within Organ Pipe Cactus National Monument), and provide a rationale for the sample choice. When relevant, describe the organism taxa, source, sex, age range and any manipulations. State what population the sample is meant to represent when applicable. For studies involving existing datasets, describe the data and its source.*
Sampling strategy	*Note the sampling procedure. Describe the statistical methods that were used to predetermine sample size OR if no sample-size calculation was performed, describe how sample sizes were chosen and provide a rationale for why these sample sizes are sufficient.*
Data collection	*Describe the data collection procedure, including who recorded the data and how.*
Timing and spatial scale	*Indicate the start and stop dates of data collection, noting the frequency and periodicity of sampling and providing a rationale for these choices. If there is a gap between collection periods, state the dates for each sample cohort. Specify the spatial scale from which the data are taken*
Data exclusions	*If no data were excluded from the analyses, state so OR if data were excluded, describe the exclusions and the rationale behind them, indicating whether exclusion criteria were pre-established.*
Reproducibility	*Describe the measures taken to verify the reproducibility of experimental findings. For each experiment, note whether any attempts to repeat the experiment failed OR state that all attempts to repeat the experiment were successful.*
Randomization	*Describe how samples/organisms/participants were allocated into groups. If allocation was not random, describe how covariates were controlled. If this is not relevant to your study, explain why.*
Blinding	*Describe the extent of blinding used during data acquisition and analysis. If blinding was not possible, describe why OR explain why blinding was not relevant to your study.*

Did the study involve field work? ☐ Yes ☐ No

Field work, collection and transport

Field conditions	*Describe the study conditions for field work, providing relevant parameters (e.g. temperature, rainfall).*
Location	*State the location of the sampling or experiment, providing relevant parameters (e.g. latitude and longitude, elevation, water depth).*
Access & import/export	*Describe the efforts you have made to access habitats and to collect and import/export your samples in a responsible manner and in compliance with local, national and international laws, noting any permits that were obtained (give the name of the issuing authority, the date of issue, and any identifying information).*
Disturbance	*Describe any disturbance caused by the study and how it was minimized.*

Reporting for specific materials, systems and methods

We require information from authors about some types of materials, experimental systems and methods used in many studies. Here, indicate whether each material, system or method listed is relevant to your study. If you are not sure if a list item applies to your research, read the appropriate section before selecting a response.

Materials & experimental systems

n/a	Involved in the study	
☐	☒	Antibodies
☐	☒	Eukaryotic cell lines
☒	☐	Palaeontology and archaeology
☐	☒	Animals and other organisms
☒	☐	Human research participants
☒	☐	Clinical data
☒	☐	Dual use research of concern

Methods

n/a	Involved in the study	
☐	☒	ChIP-seq
☐	☒	Flow cytometry
☒	☐	MRI-based neuroimaging

Antibodies

Antibodies used

Anti-ERα rabbit polyclonal (C1355) from Millipore (Billerica, MA)
Rabbit polyclonal antibody against ERα (A300-498A) from Bethyl Laboratories
Rabbit polyclonal antibody against ZEB1 (A301-921A) from Bethyl Laboratories
Rabbit polyclonal antibody against vimentin (A301-620A) from Bethyl Laboratories
Mouse monoclonal antibody against N-cadherin (13A9) from Cell Signaling Technology (#14215; Beverly, USA)
Mouse monoclonal anti-GAPDH (6C5, ab8245) from Abcam
Goat polyclonal antiserum against FOXA1 (ab5089) from Abcam
Mouse monoclonals against AP2gamma (6E4/4) Santa Cruz Biotechnology (Santa Cruz, CA, USA)
CD151 (H-8) were from Santa Cruz Biotechnology (Santa Cruz, CA, USA)
Mouse monoclonal anti-E-cadherin (C36) from BD Transduction Laboratories
Mouse BV421 anti-CD326 (EpCAM) from BD Transduction Laboratories
IgG1 k isotype control from BD Transduction Laboratories
Rabbit polyclonal against ZEB1 from Proteintech (21544-1-AP)
Rabbit polyclonal IgG (12-370) from (Sigma-Aldrich)
Rabbit polyclonal against vimentin from GeneTex (GTX100619)
Mouse EpCAM from Cell Signaling Technology (VU1D9)
HRP-conjugated anti-mouse (P044701-2) and anti-rabbit (P044801-2) secondary antibodies for immunoblotting were from Agilent Dako.
Alexa Fluor 594-conjugated AffiniPure Fab fragment from goat against rabbit IgG (H+L) (111-587-003) from Jackson ImmunoResearch Europe Ltd
Alexa Fluor 546-conjugated goat anti-mouse IgG (H+L) (A-11030) from Thermo Scientific
Alexa Fluor 488-conjugated goat anti-rabbit IgG (H+L) (A-11034) from Thermo Scientific
Alexa Fluor 488 F(ab')2-goat anti-mouse IgG (H+L) (A-11017) from Thermo Scientific

Validation

Anti-ERα rabbit polyclonal (C1355) from Millipore (Billerica, MA) validated by Western Blotting with human whole cell lysates.
Rabbit polyclonal antibody against ERα (A300-498A) from Bethyl Laboratories validated by Western Blotting, immunoprecipitation, and ChIP-seq with human whole cell lysates.
Rabbit polyclonal antibody against ZEB1 (A301-921A) from Bethyl Laboratories validated by Western Blotting and immunoprecipitation with human whole cell lysates. ChIP experiments were validated in the manuscript using control binding sites.
Rabbit polyclonal antibody against vimentin (A301-620A) from Bethyl Laboratories validated by Western Blotting with human whole cell lysates.
Mouse monoclonal antibody against N-cadherin (13A9) from Cell Signaling Technology (Beverly, USA) validated by western blotting with human whole cell lysates.
Mouse monoclonal anti-GAPDH (6C5, ab8245) from Abcam validated by Western Blotting with human whole cell lysates.
Goat polyclonal antiserum against FOXA1 (ab5089) from Abcam validated by Western Blotting with human whole cell lysates.
Mouse monoclonals against AP2gamma (6E4/4) Santa Cruz Biotechnology (Santa Cruz, CA, USA) Abcam validated by Western Blotting

with human whole cell lysates.
CD151 (H-8) were from Santa Cruz Biotechnology (Santa Cruz, CA, USA) Abcam validated by Western Blotting with human whole cell lysates.
Mouse monoclonal anti-E-cadherin (C36) from BD Transduction Laboratories Abcam validated by Western Blotting with human whole cell lysates.
Mouse BV421 anti-CD326 (EpCAM) from BD Transduction Laboratories validated by flow cytometry with human whole cell lysates.
IgG1 k isotype control from BD Transduction Laboratories validated by FACS with peripheral blood lymphocytes.
Rabbit polyclonal against ZEB1 from Proteintech (21544-1-AP) validated with various cell lines including MCF7 with western blotting, IPs, and IHC. Also validated for ChIP by various publications.
Rabbit polyclonal IgG (12-370) from (Sigma-Aldrich) validated for IP and western blotting using human breast carcinoma whole cell lysates.
Rabbit polyclonal against vimentin from GeneTex (GTX100619) validated with western blotting, IHC (Paraffin-Embedded adult mouse retina), IF (Hela cells), ELISA.
Mouse EpCAM from Cell Signaling Technology (VU1D9) validated for IF (HT-29 cells), wetern blotting, and IHC.
Alexa Fluor 594-conjugated AffiniPure Fab fragment from goat against rabbit IgG (H+L) (111-587-003) from Jackson ImmunoResearch Europe Ltd validated by FACS.
Alexa Fluor 546-conjugated goat anti-mouse IgG (H+L) (A-11030), Alexa Fluor 488-conjugated goat anti-rabbit IgG (H+L) (A-11034), and Alexa Fluor 488 F(ab')2-goat anti-mouse IgG (H+L) (A-11017) from Thermo Scientific are validated by IF in different cell lines.

Eukaryotic cell lines

Policy information about cell lines

Cell line source(s)

HEK293T and MCF7 cells were purchased from the American Type Culture Collection (ATCC). T-47D cells of the European Collection of Authenticated Cell Cultures (ECACC) were purchased from Sigma Aldrich. MCF7-V cells were a gift from Dr. Wilbert Zwart (Netherlands Cancer Institute, Amsterdam); their highly polymorphic short tandem repeat loci (STRs) were profiled using commercial services (ATCC and Microsynth) and found to be closely (88%) related to wild-type MCF7 cells; specifically, their eight core STR markers were as follows (alleles are indicated in parenthesis): D5S818 (11, 12), D13S317 (11), D7S820 (8, 9), D16S539 (11, 12), vWA (14, 15), TH01 (6), TPOX (9), and CSF1PO (10, 11).

Authentication

We used (ATCC and Microsynth) forthe authentication of MCF7-V cell line

Mycoplasma contamination

All cell lines were tested to be negative for mycoplasma

Commonly misidentified lines (See ICLAC register)

None of the cell lines used in this study were found in the commonly misidentified cell lines database

Palaeontology and Archaeology

Specimen provenance

Provide provenance information for specimens and describe permits that were obtained for the work (including the name of the issuing authority, the date of issue, and any identifying information). Permits should encompass collection and, where applicable, export.

Specimen deposition

Indicate where the specimens have been deposited to permit free access by other researchers.

Dating methods

If new dates are provided, describe how they were obtained (e.g. collection, storage, sample pretreatment and measurement), where they were obtained (i.e. lab name), the calibration program and the protocol for quality assurance OR state that no new dates are provided.

☒ Tick this box to confirm that the raw and calibrated dates are available in the paper or in Supplementary Information.

Ethics oversight

Identify the organization(s) that approved or provided guidance on the study protocol, OR state that no ethical approval or guidance was required and explain why not.

Note that full information on the approval of the study protocol must also be provided in the manuscript.

Animals and other organisms

Policy information about studies involving animals; ARRIVE guidelines recommended for reporting animal research

Laboratory animals

We used female NOD/scid GAMMA (NSG) mice, aged 10-12 weeks. NSG mice were purchased from Jackson Laboratory. Animals were kept in a standard light cycle photoperiod (12 hours light:12 hours dark, humidity: 55 % +/- 10 %, temperature: 22 +/- 2 degrees.

Wild animals

The study did not involve wild animals.

Field-collected samples

The study did not involve samples collected from the field.

Ethics oversight	All xenograft experiments were carried out in compliance with institutional and cantonal guidelines (approved mouse protocol #2781, cantonal veterinary office of Basel-City).

Note that full information on the approval of the study protocol must also be provided in the manuscript.

Human research participants

Policy information about studies involving human research participants

Population characteristics	*Describe the covariate-relevant population characteristics of the human research participants (e.g. age, gender, genotypic information, past and current diagnosis and treatment categories). If you filled out the behavioural & social sciences study design questions and have nothing to add here, write "See above."*
Recruitment	*Describe how participants were recruited. Outline any potential self-selection bias or other biases that may be present and how these are likely to impact results.*
Ethics oversight	*Identify the organization(s) that approved the study protocol.*

Note that full information on the approval of the study protocol must also be provided in the manuscript.

Clinical data

Policy information about clinical studies

All manuscripts should comply with the ICMJE guidelines for publication of clinical research and a completed CONSORT checklist must be included with all submissions.

Clinical trial registration	*Provide the trial registration number from ClinicalTrials.gov or an equivalent agency.*
Study protocol	*Note where the full trial protocol can be accessed OR if not available, explain why.*
Data collection	*Describe the settings and locales of data collection, noting the time periods of recruitment and data collection.*
Outcomes	*Describe how you pre-defined primary and secondary outcome measures and how you assessed these measures.*

Dual use research of concern

Policy information about dual use research of concern

Hazards

Could the accidental, deliberate or reckless misuse of agents or technologies generated in the work, or the application of information presented in the manuscript, pose a threat to:

No	Yes	
☒	☐	Public health
☒	☐	National security
☒	☐	Crops and/or livestock
☒	☐	Ecosystems
☒	☐	Any other significant area

Experiments of concern

Does the work involve any of these experiments of concern:

No	Yes	
☒	☐	Demonstrate how to render a vaccine ineffective
☒	☐	Confer resistance to therapeutically useful antibiotics or antiviral agents
☒	☐	Enhance the virulence of a pathogen or render a nonpathogen virulent
☒	☐	Increase transmissibility of a pathogen
☒	☐	Alter the host range of a pathogen
☒	☐	Enable evasion of diagnostic/detection modalities
☒	☐	Enable the weaponization of a biological agent or toxin
☒	☐	Any other potentially harmful combination of experiments and agents

ChIP-seq

Data deposition

☒ Confirm that both raw and final processed data have been deposited in a public database such as GEO.

☒ Confirm that you have deposited or provided access to graph files (e.g. BED files) for the called peaks.

Data access links
May remain private before publication.

The ChIP-seq data are available through the GEO accession code GSE173562 (using the editor/reviewer token: qngtycyohxyjtsl) at https://www.ncbi.nlm.nih.gov/geo/query/acc.cgi?acc=GSE173562

Files in database submission

GSM5713871	ZEB1 ChIP-seq, IgGR1
GSM5713873	ZEB1 ChIP-seq, IgGR2
GSM5713875	ZEB1 ChIP-seq, IgGR3
GSM5713877	ZEB1 ChIP-seq, ZEB1R1
GSM5713879	ZEB1 ChIP-seq, ZEB1R2
GSM5713881	ZEB1 ChIP-seq, ZEB1R3
GSM5268458	ER ChIP-seq, +DOX(Ctl)_rep1
GSM5268459	ER ChIP-seq, +DOX(Ctl)_rep2
GSM5268460	ER ChIP-seq, +DOX(Ctl)_rep3
GSM5268461	ER ChIP-seq, +DOX(Ctl)_rep4
GSM5268462	ER ChIP-seq, +DOX(E2)_rep1
GSM5268463	ER ChIP-seq, +DOX(E2)_rep2
GSM5268464	ER ChIP-seq, +DOX(E2)_rep3
GSM5268465	ER ChIP-seq, +DOX(E2)_rep4
GSM5268466	ER ChIP-seq, +DOX(FI)_rep1
GSM5268467	ER ChIP-seq, +DOX(FI)_rep2
GSM5268468	ER ChIP-seq, +DOX(FI)_rep3
GSM5268469	ER ChIP-seq, +DOX(FI)_rep4

Genome browser session
(e.g. UCSC)

"no longer applicable" for "Final submission" documents.

Methodology

Replicates

2-4 biological replicates for ChIP-seq data

Sequencing depth

A minimum of 50 million reads of paired-end sequences were used. An average of 10% of reads were mapped uniquely. Fragment lengths were 200-250 bp.

Antibodies

Rabbit polyclonal against ZEB1 from Proteintech (21544-1-AP); Rabbit polyclonal antibody against ERα (A300-498A) from Bethyl Laboratories; Rabbit polyclonal IgG (12-370) from (Sigma-Aldrich).

Peak calling parameters

Peak calling was carried out by the MACS2 tool (Version 2.1.0) of the Galaxy tool suite (https://usegalaxy.org) or by MACS2 (Version 2.1.0) with default parameters.

Data quality

The quality of the ChIP-seq data was assessed as described by the encode project (https://www.encodeproject.org/data-standards/terms/).

Software

FeatureCounts (version 2.0.0) was used to count reads per binding regions and generated a count table. The count tables were then analyzed in R with the edgeR package. Binding regions with very low number of reads were filtered out (mean of all replicates CPM < 5).

Flow Cytometry

Plots

Confirm that:

☒ The axis labels state the marker and fluorochrome used (e.g. CD4-FITC).

☒ The axis scales are clearly visible. Include numbers along axes only for bottom left plot of group (a 'group' is an analysis of identical markers).

☒ All plots are contour plots with outliers or pseudocolor plots.

☒ A numerical value for number of cells or percentage (with statistics) is provided.

Methodology

Sample preparation

The sample preparation procedure is described in detail in the method section of the manuscript.

Instrument

BD FACS Aria III Cell Sorter (BD Biosciences) and Gallios Flow Cytometer (Beckman Coulter)

Software	FlowJo
Cell population abundance	For cell cycle assays we used data acquired from a minimum of 10,000 cells. For scRNA-seq a minimum of >6,000 cells for each group was used.
Gating strategy	Cell populations were gated based on the FSC/SSC plots. For cell cycle analyses, the percentage of cells in each phase of the cell cycle was calculated based on propidium iodide staining. For scRNA-seq, an IgG1/k isotype control was used to define the gates for positive and negative cell populations.

☒ Tick this box to confirm that a figure exemplifying the gating strategy is provided in the Supplementary Information.

Magnetic resonance imaging

Experimental design

Design type	*Indicate task or resting state; event-related or block design.*
Design specifications	*Specify the number of blocks, trials or experimental units per session and/or subject, and specify the length of each trial or block (if trials are blocked) and interval between trials.*
Behavioral performance measures	*State number and/or type of variables recorded (e.g. correct button press, response time) and what statistics were used to establish that the subjects were performing the task as expected (e.g. mean, range, and/or standard deviation across subjects).*

Acquisition

Imaging type(s)	*Specify: functional, structural, diffusion, perfusion.*
Field strength	*Specify in Tesla*
Sequence & imaging parameters	*Specify the pulse sequence type (gradient echo, spin echo, etc.), imaging type (EPI, spiral, etc.), field of view, matrix size, slice thickness, orientation and TE/TR/flip angle.*
Area of acquisition	*State whether a whole brain scan was used OR define the area of acquisition, describing how the region was determined.*

Diffusion MRI ☐ Used ☐ Not used

Preprocessing

Preprocessing software	*Provide detail on software version and revision number and on specific parameters (model/functions, brain extraction, segmentation, smoothing kernel size, etc.).*
Normalization	*If data were normalized/standardized, describe the approach(es): specify linear or non-linear and define image types used for transformation OR indicate that data were not normalized and explain rationale for lack of normalization.*
Normalization template	*Describe the template used for normalization/transformation, specifying subject space or group standardized space (e.g. original Talairach, MNI305, ICBM152) OR indicate that the data were not normalized.*
Noise and artifact removal	*Describe your procedure(s) for artifact and structured noise removal, specifying motion parameters, tissue signals and physiological signals (heart rate, respiration).*
Volume censoring	*Define your software and/or method and criteria for volume censoring, and state the extent of such censoring.*

Statistical modeling & inference

Model type and settings	*Specify type (mass univariate, multivariate, RSA, predictive, etc.) and describe essential details of the model at the first and second levels (e.g. fixed, random or mixed effects; drift or auto-correlation).*
Effect(s) tested	*Define precise effect in terms of the task or stimulus conditions instead of psychological concepts and indicate whether ANOVA or factorial designs were used.*

Specify type of analysis: ☐ Whole brain ☐ ROI-based ☐ Both

Statistic type for inference (See Eklund et al. 2016)	*Specify voxel-wise or cluster-wise and report all relevant parameters for cluster-wise methods.*
Correction	*Describe the type of correction and how it is obtained for multiple comparisons (e.g. FWE, FDR, permutation or Monte Carlo).*

Models & analysis

n/a	Involved in the study
☐	☐ Functional and/or effective connectivity
☐	☐ Graph analysis
☐	☐ Multivariate modeling or predictive analysis

Functional and/or effective connectivity	*Report the measures of dependence used and the model details (e.g. Pearson correlation, partial correlation, mutual information).*
Graph analysis	*Report the dependent variable and connectivity measure, specifying weighted graph or binarized graph, subject- or group-level, and the global and/or node summaries used (e.g. clustering coefficient, efficiency, etc.).*
Multivariate modeling and predictive analysis	*Specify independent variables, features extraction and dimension reduction, model, training and evaluation metrics.*
